# Non-parsimonious evolution of hagfish *Dlx* genes

**DOI:** 10.1186/1471-2148-13-15

**Published:** 2013-01-19

**Authors:** Satoko Fujimoto, Yasuhiro Oisi, Shigehiro Kuraku, Kinya G Ota, Shigeru Kuratani

**Affiliations:** 1 Laboratory for Evolutionary Morphology, RIKEN Center for Developmental Biology, Kobe, 650-0047, Japan; 2 Department of Biology, Graduate School of Science, Kobe University, Kobe, Japan; 3 Genome Resource and Analysis Unit, RIKEN Center for Developmental Biology, Kobe, 650-0047, Japan; 4 Laboratory of Aquatic Zoology, Marine Research Station, Institute of Cellular and Organismic Biology, Academia Sinica, Yilan, 26242, Taiwan

**Keywords:** Cyclostomes, Hagfish embryos, *Dlx* genes, Gene duplication

## Abstract

**Background:**

The number of members of the *Dlx* gene family increased during the two rounds of whole-genome duplication that occurred in the common ancestor of the vertebrates. Because the *Dlx* genes are involved in the development of the cranial skeleton, brain, and sensory organs, their expression patterns have been analysed in various organisms in the context of evolutionary developmental biology. Six *Dlx* genes have been isolated in the lampreys, a group of living jawless vertebrates (cyclostomes), and their expression patterns analysed. However, little is known about the *Dlx* genes in the hagfish, the other cyclostome group, mainly because the embryological analysis of this animal is difficult.

**Results:**

To identify the hagfish *Dlx* genes and describe their expression patterns, we cloned the cDNA from embryos of the Japanese inshore hagfish *Eptatretus burgeri*. Our results show that the hagfish has at least six *Dlx* genes and one pseudogene. In a phylogenetic analysis, the hagfish *Dlx* genes and those of the lampreys tended to be excluded from the clade of the gnathostome *Dlx* genes. In several cases, the lamprey *Dlx* genes clustered with the clade consisting of two hagfish genes, suggesting that independent gene duplications have occurred in the hagfish lineage. Analysis of the expression of these genes showed distinctive overlapping expression patterns in the cranial mesenchymal cells and the inner ear.

**Conclusions:**

Independent duplication, pseudogenization, and loss of the *Dlx* genes probably occurred in the hagfish lineage after its split from the other vertebrate lineages. This pattern is reminiscent of the non-parsimonious evolution of its morphological traits, including its inner ear and vertebrae, which indicate that this group is an early-branching lineage that diverged before those characters evolved.

## Background

The extant vertebrates are divided into two major groups, the jawed (gnathostomes) and the jawless vertebrates (agnathans). The two groups share a number of morphological characters (synapomorphies) that define the vertebrates, such as the neurogenic placode, neural crest, and their derivatives, including complex sense organs and a cranial skeleton [1-3]. These morphological characters are not seen in non-vertebrate chordates. To investigate the early phase of vertebrate evolution from a molecular perspective, the expression patterns of various developmental regulatory genes have been compared between the gnathostomes and the lamprey, one of the two extant groups of agnathans [4-14]. In contrast to the lamprey, little is known about the developmental processes of these morphological characters in the hagfish because their embryos have been unavailable until recently.

The cyclostomes are often recognized as a paraphyletic group in the fields of morphology and palaeontology [3,15-17] because of the extraordinarily different morphologies of the hagfish and lampreys [18,19]. In fact, the hagfish has been considered to lack a number of the vertebrate characters possessed by the lamprey, such as de-epithelialized and migrating neural crest cells, vertebral elements, a complex branchial basket, and multiple semicircular canals in the inner ear [18-22]. Based on the idea that these relatively simple morphological features of the hagfish represent the ancestral state of the vertebrates, this animal has tended to be placed at the base of the phylogenetic tree of the entire vertebrates [3,15-17]. However, on various molecular phylogenetic trees, the hagfish tends to cluster with the lamprey in a monophyletic group, and this position is now widely accepted by researchers who are familiar with these molecular phylogenetic analyses [23-27]. This discrepancy between the molecular and morphological data has been a source of contention regarding the evolution of the early vertebrates, and there was no consensus on the phylogenetic position of the hagfish for about three decades [28].

**Figure 1:**
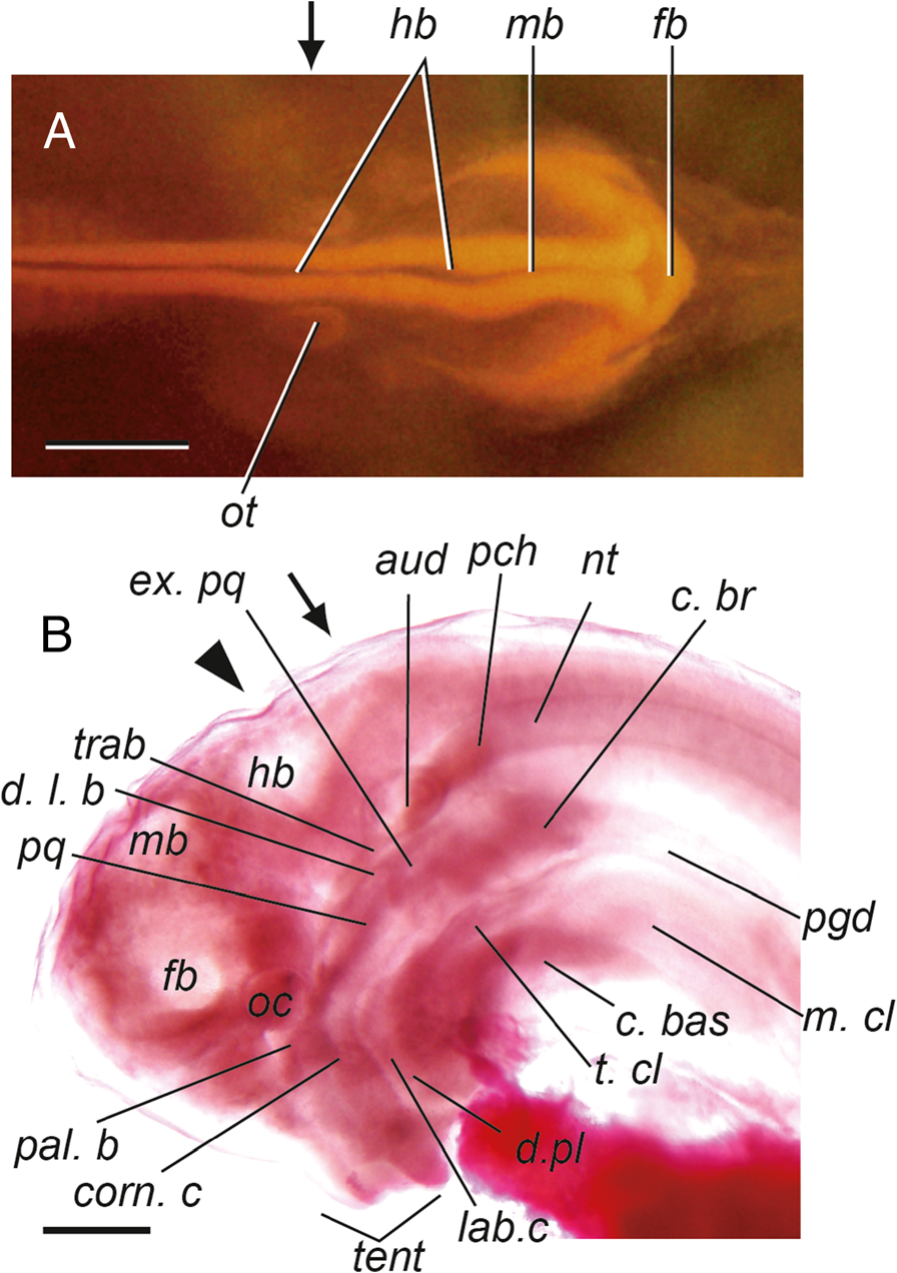
**Head regions of middle- and late-pharyngula embryos of *Eptatretus burgeri. ***(**A**) Dorsal view of the middle-pharyngula embryo. (**B**) Lateral view of the late-pharyngula embryo. The levels of the sections used in the histological analysis and *in situ* hybridization are shown by arrows (otic level) and arrowheads (pre-otic levels). Abbreviations: *aud*, auditory capsule; *c. br*, branchial cartilage; *c. bas*, basal cartilage; *d. l. b*, dorsal longitudinal bar; *d. pl*, dental plate; *ex. pq*, extrapalato-quadrate; *fb*, forebrain; *hb*, hindbrain; *lab. c*, labial cartilage; *m. cl*, clavatus muscle; *mb*, midbrain; *nt*, notochord; *oc*, eye; *ot*, otic vesicles; *pal. b*, paraline bar; *pch*, prechordal plate; *pq*, palato-quadrate; *t. cl*, tendon of the clavatus muscle; *tent*, tentacles; *trab*, trabecula. Scale bar: 500 μm.

However, in this century, the situation in the field of hagfish research has changed. Since 2007, a number of live embryos of the Japanese inshore hagfish, *Eptatretus burgeri*, have been obtained (Figure 1), and these have provided an opportunity to conduct intensive molecular cloning and analysis of the hagfish embryonic gene expression patterns [29-31]. Even more significantly, these molecular studies of hagfish embryos have demonstrated that, contrary to the suggestions of traditional text-books, the hagfish has de-epithelialized and migrating neural crest cells and vertebral elements [19,30-33]. Furthermore, a comparative analysis of the hagfish and lamprey microRNAs tended to support a monophyletic relationship between them [34]. These lines of evidence seem to have established a consensus among molecular biologists and palaeontologists on the phylogenetic position of the hagfish. In fact, in a recently published paleontological report on the evolution of the early vertebrates, the hagfish is recognized as the sister group of the lampreys [35]. Therefore, it is now possible to reconstruct a more plausible evolutionary scenario of how the morphology of the hagfish diverged from that of the other vertebrates, by examining the embryonic events of this animal with molecular biological techniques.

Given the monophyly of the cyclostomes, it is conceivable that the molecular developmental mechanisms of the lampreys and hagfish evolved independently in each lineage after their divergence more than 400 million years ago [36], resulting in secondarily degenerate characters that are more marked in the hagfish lineage. In fact, this assumption is consistent, at the molecular level, with the evidence that the *Xlox* gene, one of the ParaHox genes responsible for organogenesis (including pancreas formation) in the gnathostomes, is pseudogenized in the genome of the Atlantic hagfish (*Myxine glutinosa*), which may correlate with the absence of some endocrine organs in the hagfish [37]. This suggests that some molecular sequences and expression patterns could be secondarily degenerate for other hagfish genes that are involved in the developmental processes of the vertebrate morphological synapomorphies.

Because the *Dlx* genes are crucial to the morphogenesis of the vertebrate synapomorphies, they may also be secondarily degenerate in the hagfish [38-42]. The *Dlx* genes, homeobox-containing transcription factors, are organized in convergently transcribed bi-gene clusters, which are linked to the *Hox* gene clusters in the genomes of the gnathostomes. For example, the six *Dlx* genes of mammals form three bi-gene clusters, *Dlx1Dlx2*, *Dlx3Dlx4*, and *Dlx5Dlx6*, linked to the *HoxD*, *HoxB*, and *HoxA* clusters, respectively [43-47]. From the evidence that the chondrichthyan species have three *Dlx* bi-gene clusters, it is presumed that the common ancestor of the gnathostomes already had three bi-gene clusters, which seem to have derived from two rounds of genome duplication [44,48]. More significantly, the *Dlx* genes show overlapping expression patterns in the synapomorphic characters of the gnathostomes; for example, in the forebrain, neural crest cells, and inner ear [40-42]. Almost all the *Dlx* genes are expressed in the ectomesenchymal cells derived from the cranial neural crest [41,49,50], and the expression of some *Dlx* genes is detected during the developmental processes of the inner ear [40,42] in model gnathostome species, including the chicken, mouse, and zebrafish.

It has been demonstrated that the lamprey has at least six *Dlx* genes, designated *DlxAF*[13,51]. At least four of these genes were generated by independent duplications unique to the lineage of either the lampreys or the cyclostomes. It is also known that *DlxA–E* display overlapping expression patterns in the cranial ectomesenchyme and some of them in the otic vesicles [7,13,14]. These data raise several simple questions. How many *Dlx* genes are there in the hagfish genome? Did the hagfish ancestor also undergo an independent duplication of the *Dlx* genes, as in the lamprey lineage?

The derivatives of the cranial ectomesenchyme and the otic vesicle are also clearly simpler in the hagfish than in the lampreys and gnathostomes at the morphological level [18,30]. For example, the basket-like structure of the branchial skeleton is present in the lampreys but absent in the hagfish. There is also no vertical semicircular canal in the hagfish inner ear [18,19]. These lines of evidence raise another question. Do the *Dlx* genes of the hagfish also show overlapping expression patterns in the cranial ectomesenchyme and otic vesicles, similar to those in the lamprey and gnathostomes?

To address these questions, we cloned the *Dlx* genes from embryonic materials of the Japanese inshore hagfish (*E. burgeri*) and analysed the gene expression patterns of the isolated hagfish *Dlx* genes using *in situ* hybridization. Here, we show that the hagfish has at least six *Dlx* genes, some of which arose from gene duplications unique to the hagfish lineage. Furthermore, some of the isolated hagfish *Dlx* genes are expressed in the cranial mesenchymal cells and otic vesicles with overlapping expression patterns, as reported in the lampreys and gnathostomes [6,7,13,41,42]. These results suggest that the *Dlx* genes were independently duplicated and then diverged in each of the hagfish and lamprey lineages, maintaining overlapping expression patterns.

## Results

### Identification of hagfish *Dlx*-encoding cDNAs

**Figure 2:**
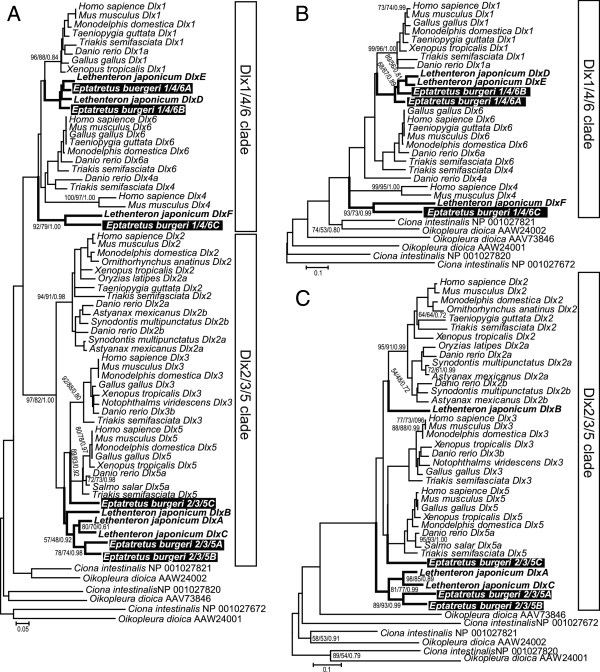
**Molecular phylogenetic trees including the hagfish *Dlx *genes. **The topologies and branch lengths were calculated with the neighbour-joining method. (**A**) Phylogenetic tree containing the *Dlx1/4/6* and *Dlx2/3/5* genes (based on 77 amino acid sites). (**B**) Phylogenetic tree of *Dlx1/4/6* (based on 101 amino acid sites). (**C**) Phylogenetic tree of *Dlx2/3/5* (based on 102 amino acid sites). Bootstrap probabilities (NJ and ML) greater than 50 and posterior probabilities (BI) greater than 0.5 are shown at each internal branch supported by all three methods, as the left, middle, and right numbers, respectively. The clades of the cyclostome *Dlx* genes are indicated with bold lines. The hagfish genes are shown in white bold letters in black boxes.

To isolate full-length cDNAs containing the *Dlx* genes from the hagfish, we used degenerate RT–PCR and 5^′^/3^′^ RACE-PCR to amplify seven candidate genes from the embryonic material of *E. burgeri* (see Methods). All these isolated cDNAs contained nucleotide stretches corresponding to homeodomains, and one of them had a stop codon in the homeodomain (see Additional file 1). In our sequence comparison and preliminary molecular phylogenetic analysis, these genes were classified into two major groups (the *Dlx1/4/6* and *Dlx2/3/5* clades; Figure 2A). Because of the paucity of informative amino acid sites and the unstable positions of the outgroups, we could not identify the strict orthology of the isolated hagfish genes with the gnathostome *Dlx* genes. Therefore, we categorized and designated these seven genes from *E. burgeri EbDlx1/4/6A*, *EbDlx1/4/6B*, *EbDlx1/4/6C*, *EbDlx2/3/5A*, *EbDlx2/3/5B*, *EbDlx2/3/5C*, and *EbDlxΨ*, according to the sequence similarities of the homeodomains and their flanking regions and the topology of the phylogenetic tree.

From a comparison of the conserved regions of the amino acid sequences, it is expected that *EbDlxΨ* and *EbDlx2/3/5B* are closely related to each other, implying that they diverged recently in the hagfish lineage. However, we could not identify the phylogenetic position of this pseudogene from our data set, because of the paucity of informative amino acid site between *EbDlxΨ* and the other sequences (see Additional file 1). Therefore, we excluded *EbDlxΨ* from our phylogenetic analysis.

### Molecular phylogeny of the hagfish *Dlx* genes

Molecular phylogenetic trees of the vertebrate *Dlx* genes were reconstructed using their homologues in two tunicate species (*Ciona intestinalis* and *Oikopleura dioica*) as outgroups. Although the positions of the tunicate *Dlx* homologues were not stable, these genes were excluded from the clades of the gnathostome and cyclostome *Dlx* genes in our phylogenetic trees (Figure 2). These outgroups are positioned on the internal branch connecting the *Dlx1/4/6* and *Dlx2/3/5* gene clades of the vertebrates on a phylogenetic tree consisting of 68 operational taxonomic units (Figure 2A). On this phylogenetic tree, *EbDlx1/4/6C* forms a cluster with *DlxF* of the lamprey with strong support, *EbDlx2/3/5A* and *EbDlx2/3/5B* cluster in a clade containing three lamprey genes (*DlxA*, -*B*, and -*C*), and *EbDlx1/4/6A* and -*B* cluster with two lamprey genes (*DlxD* and *-E*) (Figure 2A). Exceptionally, *EbDlx2/3/5C* was isolated from the other cyclostome *Dlx* genes on this phylogenetic tree.

To increase the number of informative amino acid sites for the phylogenetic analysis and to improve the resolution of the phylogenetic tree, we separately analysed the *Dlx1/4/6* and *Dlx2/3/4* clade genes with/without the outgroups (Figure 2B, C; Additional File 2). The clade of *EbDlx1/4/6C* and the lamprey *DlxF* gene was located at the most basal position of the vertebrate *Dlx1/4/6* gene clade on the phylogenetic tree that included the outgroups (Figure 2B). To maximize the number of informative amino acid sites used in the phylogenetic inference, we excluded the sequences that produced extremely long branches (all tunicate *Dlx* homologues, gnathostome *Dlx4* and −6, *EbDlx1/4/6C*, and lamprey *DlxF* genes) from the alignment. On this phylogenetic tree, four *Dlx* genes of the cyclostomes formed a single clade, in which two *EbDlx1/4/6* genes clustered with a high bootstrap value (Additional file 2A). The cluster containing the lamprey *DlxD* and *DlxE* genes was located next to the hagfish cluster, although the bootstrap value was not very high. This phylogenetic tree indicates that the duplication that produced *EbDlx1/4/6A* and *EbDlx1/4/6B* occurred in the hagfish lineage after its separation from the lamprey lineage.

The tree topology of the *Dlx2/3/5* subfamily also suggested an independent gene duplication in the hagfish lineage (Figure 2C; Additional file 2). The cluster containing *EbDlx2/3/5A* and *EbDlx2/3/5B* formed a sister group with the cluster containing lamprey *DlxA* and *DlxC*, with high bootstrap support. This cluster was located at the basal position of the *Dlx2/3/5* clade (Figure 2C). In the phylogenetic tree without outgroups, the clade containing *EbDlx2/3/5A*, *EbDlx2/3/5B*, lamprey *DlxA*, and *DlxC* had a high supporting value in all the phylogenetic methods used (Additional file 2), suggesting that independent gene duplications had occurred in both the lamprey and hagfish lineages. This phylogenetic tree also suggested that the *EbDlx2/3/5C* and lamprey *DlxB* genes are most closely related to each other (Additional file 2).

### Embryonic expression patterns of the hagfish *Dlx* genes

**Figure 3:**
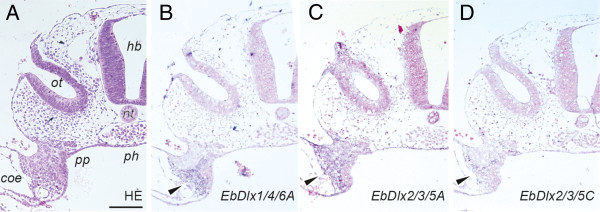
**Expression patterns of the *Dlx *genes in the middle-pharyngula embryo at the otic placode level. **(**A**) Transverse view of a haematoxylin- and eosin-stained section. (**B**–**D**) Expression patterns of the *Dlx* genes. DIG-labelled probes for *EbDlx1/4/6A* (**B**), *EbDlx2/3/5B* (**C**), and *EbDlx2/3/5C* (**D**) were detected in the ectomesenchymal cells (arrowheads). The sections used for *in situ* hybridization were counterstained with ISH Red (**B**–**D**). The level of the section is indicated by an arrow in Figure 1A. Abbreviations: *coe*, coelom; *hb*, hindbrain; *nt*, notochord; *ot*, otic vesicles; *ph*, pharynx; *pp*, pharyngeal pouch. Scale bar, 100 μm.

**Figure 4:**
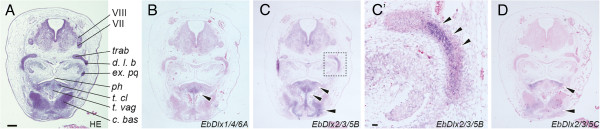
**Expression patterns of the *Dlx *genes in the late-pharyngula embryo at the inner ear level. **(**A**) Transverse view of a haematoxylin- and eosin-stained section. (**B**–**D**) Expression patterns of the *Dlx* genes. The sections for *in situ* hybridization were counterstained with ISH Red (**B**–**D**). (**B**) Expression of *EbDlx1/4/6A* was detected in the mesenchymal cells at *t. vag* (arrowhead). (**C**) *EbDlx2/3/5B* showed a broad expression pattern in three skeletal elements (*t. cl*, *t. vag*, and *c. bas*) (arrowheads). (**C**^′^) Higher magnification of the dotted box shown in **C**; restricted expression pattern of *EbDlx2/3/5B* was detected in one cartilaginous complex consisting of *d. l. b*, *ex. pq*, and *trab*, (arrowheads). (**D**) *EbDlx2/3/5C* was expressed in the *c. bas* and mesenchymal cells on the ventral aspect of *ph*. The level of the section is indicated by an arrowhead in Figure 1B. Abbreviations: *c. bas*, basal cartilage; *d. l. b*, dorsal longitudinal bar; *ex. pq*, extrapalato-quadrate; *ph*, pharynx; *t. cl*, tendon of the clavatus muscle; *t. vag*, tendon of the vagina of the clavatus; *trab*, trabecula; VII, facialis nerve; VIII, saccularis nerve. Scale bars, 100 μm (**A**); 10 μm (**C**^′^).

**Figure 5:**
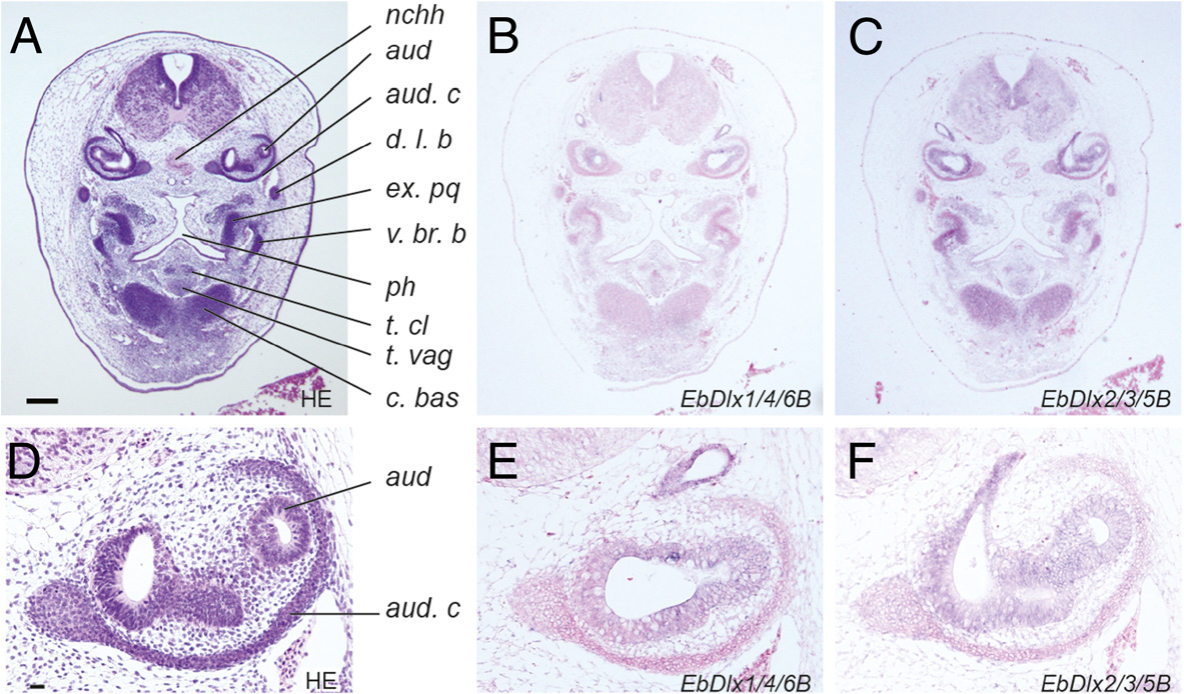
**Expression patterns of the *Dlx *genes in the late-pharyngula embryo at the inner ear level. **(**A**) Transverse view of a haematoxylin- and eosin-stained section. (**B**, **C**) Expression patterns of the *Dlx* genes. The sections for *in situ* hybridization were counterstained with ISH Red (**B**, **C**, **E**, **F**). (**D**–**F**) Higher magnification of the inner ear regions in **A**–**C**, respectively. *EbDlx1/4/6B* and *EbDlx2/3/5B* were weakly expressed in *aud* (**E**, **F**). The level of the section is indicated by an arrow in Figure 1B. Abbreviations: *aud*, auditory capsule; *aud. c*, auditory cartilage; *c. bas*, basal cartilage; *d. l. b*, dorsal longitudinal bar; *ex. pq*, extrapalato-quadrate; *nchh*, notochordal hook; *ph*, pharynx; *t. cl*, tendon of the clavatus muscle; *t. vag*, tendon of the vagina of the clavatus; *v. br. b*, ventral branchial bar. Scale bars, 100 μm (**A**); 10 μm (**D**).

To observe the expression patterns of the hagfish *Dlx* genes in the primordia of the cranial skeleton and inner ear, we used *in situ* hybridization in the head regions of hagfish embryos at two different stages (designated “middle-” and “late-pharyngula embryos”; Figure 1). In these two embryos, we conducted anatomical observations and identified the exact locations of the otic and optic vesicles and several skeletal tissues, with reference to the descriptions and sketches of previous researchers, including Dean (1899) [52], Cole (1905) [20], and Holmgren (1973) [53]. We examined transverse sections at the antero-posterior level containing the otic vesicle, the rostral pharyngeal pouches, and the head mesenchyme in the middle-pharyngula embryo, and at the levels of the otic to pre-otic regions in the late-pharyngula embryo, for the expression of all six hagfish *Dlx* genes and the pseudogene (Figures 1, 3, 4 and 5; see also Additional file 3).

In the middle-pharyngula embryo, transcripts of *EbDlx1/4/6A*, *EbDlx2/3/5A*, and *EbDlx2/3/5C* were detected in the mesenchymal cells between the pharyngeal endoderm and the surface ectoderm, which are presumptive cranial neural crest cells (Figure 3). Although not as distinct as the expression patterns of those three genes, *EbDlx1/4/6B* and *EbDlx2/3/5B* tended to be detected in the lateral ventral part of the epithelial cells of the otic vesicles (Additional file 3). In the late-pharyngula embryo at the pre-otic level, which contains the trabecular cartilage (indicated by the arrowhead in Figure 1B; Figure 4A), transcripts of *EbDlx1/4/6A*, *EbDlx2/3/5B*, and *EbDlx2/3/5C* were detected (Figure 4B-D). The signal for *EbDlx1/4/6A* was detected in the mesenchymal cells of the primordium of the tendons of the vagina of the clavatus, which is one of the tissues of the hagfish feeding apparatus (Figure 4B). The expression of *EbDlx2/3/5B* was observed in different mesenchymal cells of the primordial tendons and cartilages, including the tendons of the clavatus muscle and the vagina of the clavatus, the basal cartilage, and the cartilage that comprises the trabecula, dorsal longitudinal bar, and extrapalato-quadrate (Figure 4C). A highly intense *EbDlx2/3/5C* signal was detected in the mesenchyme located on the ventral aspects of the pharynx and the basal cartilage (Figure 4D). At the level of the inner ear (Figure 1B, arrow), *EbDlx1/4/6B* and *EbDlx2/3/5B* showed distinct expression patterns in the auditory capsule (Figure 5). *EbDlx2/3/5B* was expressed broadly throughout the auditory capsule with homogeneous intensity (Figure 5B, E), whereas *EbDlx1/4/6B* showed a strong signal on the lateral side of the auditory capsule (Figure 5C, F).

The expression pattern of *EbDlxΨ* is broad, and no specific signals were detected in the middle-pharyngula stage. In the late-pharyngula stage, this pseudogene showed specific expression patterns in the cranial cartilages and inner ear (Additional file 3).

## Discussion

In this study, we successfully isolated seven hagfish cDNAs, whose deduced amino acid sequences show significant similarity to the Dlx sequences of the jawed vertebrates. Our molecular phylogenetic analyses suggested that six of these with intact open reading frames are homologues of the *Dlx* genes reported for other vertebrates, and the other is a transcribed pseudogene with a nonsense nucleotide substitution. We also analysed the expression patterns of all six isolated genes in middle- and late-pharyngula hagfish embryos. From our results, we deduced the common ancestral state and evolutionary processes of the *Dlx* genes in the cyclostome lineage, based on our previous knowledge of the molecular phylogeny and gene expression patterns of the *Dlx* genes of the lampreys and gnathostomes [7,13,14,38-42].

**Figure 6:**
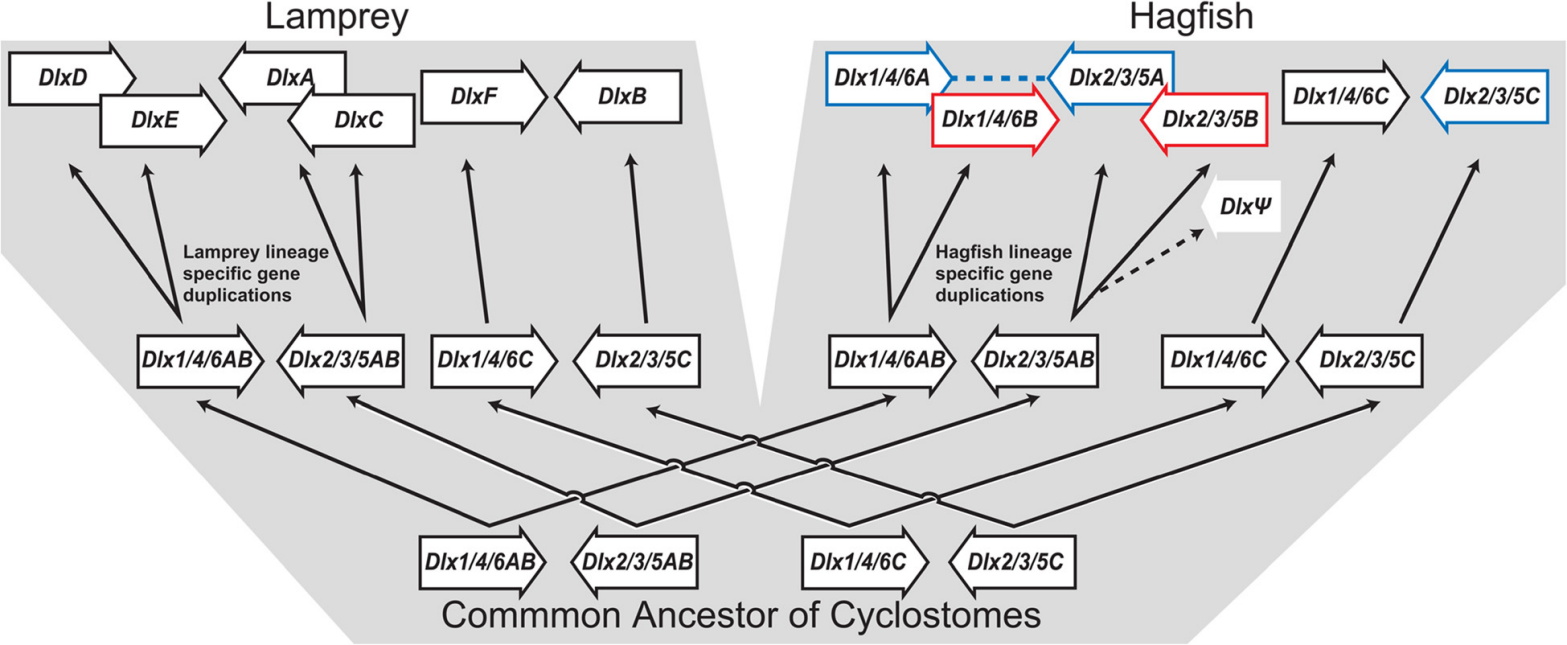
**Hypothetical scheme of the expansion of the *Dlx *genes in the cyclostome lineage. **The right- and left-pointing boxed arrows represent the *Dlx1/4/6* and −*2/3/5* clade genes. Red and blue boxed arrows indicate the *Dlx* genes, showing the overlapping expression patterns in the inner ear and cranial ectomesenchymal cells, respectively. The grey area shows the species tree of the hagfish and lampreys. Black arrows indicate the evolutionary processes of the *Dlx* genes. The pseudogene and its evolutionary process are indicated by a white arrow and dotted line, respectively.

Although the phylogenetic relationships among all the *Dlx* genes are not fully resolved, the topologies of the phylogenetic trees indicate that some of the hagfish and lamprey *Dlx* genes were duplicated in the cyclostome lineage (Figures 2 and 6). Because *EbDlx1/4/6A* and -*B* and *EbDlx2/3/5A* and -*B* form clusters with high bootstrap support, it seems plausible that these genes were duplicated uniquely in the hagfish lineage (Additional file 2). The same assumption can be made for the *DlxA*, -*C*, -*D*, and -*E* genes of the lamprey, as shown previously [51]. Thus, the evolution of these cyclostome *Dlx* genes can be described as follows. First, the *Dlx1/4/6AB*, *Dlx2/3/5AB*, *Dlx1/4/6C*, and *Dlx2/3/5C* genes existed in the genome of the last common ancestor of the hagfish and lampreys (Figure 6; the boxed arrows labelled *Dlx1/4/6AB, Dlx2/3/5AB*, *Dlx1/4/6C*, and *Dlx2/3/5C*). Second, at the divergence of the two cyclostome lineages, the hagfish and lampreys, these ancestral cyclostome *Dlx* genes were segregated into the two species lineages (Figure 6; the boxed arrows labelled *Dlx1/4/6AB*, *Dlx2/3/5AB*, and *Dlx2/3/5C*). Third, independent gene duplications in the hagfish and lamprey lineages produced two additional *Dlx* genes in each lineage (Figure 6; the boxed arrows labelled *DlxA*, *DlxC*, *DlxD*, *DlxE*, *Dlx1/4/6A*, *Dlx2/3/5A*, *Dlx1/4/6B*, and *Dlx2/3/5B*). To confirm this hypothetical scenario, several problems must be resolved. One is our confidence in the phylogenetic relationships among the cyclostome *Dlx* genes. We cannot completely exclude the possibility that the *Dlx* genes were duplicated at a more ancient point in time, such as in the common ancestor of the cyclostomes. It has previously been suggested that the unique sequence characters of the lamprey genes probably resulted in the exclusive lamprey gene clusters on the phylogenetic trees, erroneously supporting lamprey-lineage-specific gene duplications [52]. This may also have occurred in the hagfish genes.

It also remains to be ascertained whether the bi-gene cluster structure of the *Dlx* genes has been conserved in the cyclostomes during the entire course of their evolution. Given that the bi-gene clusters were already present before the divergence of the cyclostomes and gnathostomes [44,51,54,55], it is reasonable to assume that the ancestral cyclostome *Dlx* genes were also present in bi-gene clusters (Figure 6). Our data show that the expression domains of the hagfish *Dlx* genes overlap (Figures 3 and 5). The expression of *EbDlx1/4/6A*, *EbDlx2/3/5A*, and *EbDlx2/3/5C* was detected in the pharyngeal mesenchymal cells of the middle-pharyngula embryo (Figure 3), as is seen in the lampreys and gnathostomes [7,13,14]. A number of conserved *cis*-acting regulatory elements that are shared by two different *Dlx* genes and contribute to their overlapping expression patterns in the pharyngeal mesenchymal cells have been identified in the intergenic regions of the gnathostome *Dlx* gene clusters [56-58]. Therefore, it seems plausible that the hagfish *Dlx* genes that show overlapping expression patterns in the pharyngeal mesenchymal cells have retained the ancestral bi-gene clusters, with conservation of these *cis*-acting regulatory elements (the blue boxed arrows and the dashed line in Figure 6).

In this context, it is worth considering the evolutionary relationships between the two rounds of whole-genome duplication, the number of *Dlx* genes, and their linkage to other genes. Based on the presumption that the *Hox* cluster and its associated genes (including the *Dlx* genes), the so-called the “core-Hox paralogon”, were increased by the two rounds of genomic duplication in the common ancestor of the vertebrates [46,47], the two *Dlx* genes in each bi-gene cluster are also thought to have been duplicated twice, consequently producing eight *Dlx* genes in total, including the two hypothetical genes (*Dlx7* and *Dlx8*) in the genome of the common vertebrate ancestor. To explain why sharks and mammals have only six *Dlx* genes (*Dlx1–6*), it is assumed that the two hypothetical *Dlx* genes (*Dlx7* and *Dlx8*) that should have been linked to the *HoxC* cluster and *Col2A1* were lost before the radiation of the extant gnathostomes [44]. This raises the question of whether this loss predated the split of the cyclostome lineage from the gnathostome lineage and, if not, whether the genomes of the modern cyclostome species have retained *Dlx7* and *Dlx8*. To answer this question, further investigation of their whole genomes is required, with particular consideration of whether the *Dlx* genes are localized in the genomic regions containing the *HoxC* cluster and *Col2A1* genes in the cyclostome genomes. To investigate all these issues, the whole-genome sequence of the hagfish is required.

Recent progress in phylogenetic analysis has allowed us to construct reliable phylogenetic trees, and these trees support the monophyly of the cyclostomes [25-27,34,59]. However, identifying the orthology and resolving the molecular phylogeny of many cyclostome genes remain challenging [60]. In fact, our study has shown that the evolutionary processes of the hagfish *Dlx* genes cannot be explained by any simple parsimonious assumption based on a one-to-one orthology between the hagfishes, lampreys, and gnathostomes. Non-parsimonious evolutionary processes with “hidden paralogy”, involving ancient gene duplications followed by lineage-specific gene losses, have been proposed for several genes, including *Cdx* and *Bmp2/4/16*[61]. Therefore, we must be cautious in applying the parsimonious assumption to the evolutionary processes of other cyclostome genes. This caution also seems applicable to the hagfish morphological characters, such as the inner ear and cranial skeleton [19,30], in which the embryonic function of the *Dlx* genes is implicated, although these morphological characters have long been interpreted as plesiomorphic in the classical “Vertebrata”, excluding the hagfishes, based on parsimonious assumptions in the field of palaeontology [15-17].

## Conclusions

Our study has shown that the hagfish has retained at least six intact protein-coding *Dlx* genes and a single pseudogene. Conventional molecular phylogenetic methods suggest that four of these were generated by independent gene duplications in the hagfish lineage. These data indicate that more than half the *Dlx* genes of the hagfish are paralogous to the *Dlx* genes of the lampreys and gnathostomes, suggesting a complex gene phylogeny, possibly involving lineage-specific gene losses. The hagfish *Dlx* genes show overlapping embryonic expression patterns, as previously observed in the lampreys and gnathostomes. Our data indicate that the evolutionary processes of the hagfish *Dlx* genes cannot be explained by a simple evolutionary scenario inferred according to the principle of maximum parsimony.

## Methods

### Sample collection, aquarium maintenance, and embryonic materials

Adult male and female *E. burgeri* were collected in the Japan Sea off Shimane and Yamaguchi. The male and female individuals were maintained in an aquarium tank and a cage in the sea according to published methods [29,30,32]. From 2008 to 2009, 42 hagfish embryos were obtained in the aquarium tank and the cage. We gave identification numbers to these embryos [31] and staged them according to Dean’s figures and descriptions [52]. Among these embryos, the middle- and late-pharyngula stage embryos (#0903 and #B04, respectively) were selected as the most appropriate embryos for histological analysis and the analysis of the expression patterns of the *Dlx* genes.

### Molecular cloning

Molecular cloning and sequencing were performed according to a previous report [30]. A single pharyngula-stage embryo, which was obtained in 2007 [30], was used for total RNA extraction by using TRIzol Reagent (Invitrogen, Japan). Degenerate RT–PCR was performed to amplify the fragment of cDNA encoding the conserved homeobox region of the Dlx amino acid sequence, using three different degenerate primer sets (Additional file 4). The PCR products were isolated on a 2% agarose gel, and the individual bands were excised from the gel. The amplified PCR fragments containing the conserved homeobox regions of the *Dlx* genes were independently ligated with the TOPO TA Cloning Dual Promoter Kit (Invitrogen) and transformed into *Escherichia coli* DH5α. In total, more than 12 clones were picked from each population of clones and sequenced using an ABI 3130XL automated sequencer (Applied Biosystems, Japan). To determine the full-length cDNA sequences, the 5′ and 3′ ends were amplified with a GeneRacer kit (Invitrogen) with specific primers (Additional file 4) and sequenced with the method described above. Sequence traces were aligned with CodonCode Aligner (CodonCode Corporation, Dedham, MA, USA). To confirm the stop codon site in *EbDlxΨ*, PCR and sequencing were performed with three different cDNA samples, which were isolated from different hagfish embryos, using specific primers (Additional file 4). The sequence data were submitted to the DDBJ database [DDBJ:AB679710–AB679716].

### Molecular phylogenetic analysis

A multiple sequence alignment of the *Dlx* genes derived from representative vertebrate species was constructed with the CLUSTALW multiple alignment program [62] (also see Additional file 1) and refined by visual inspection. Based on this alignment, molecular phylogenetic analyses were performed using three different methods: the neighbour-joining (NJ), maximum likelihood (ML), and Bayesian inference (BI) methods. The NJ and ML trees were constructed with 1000 bootstrap replications. JTT and WAG models were used to construct the NJ and ML trees, respectively. The BI analyses were based on two independent runs of two million generations, with samples taken from every 100 generations. Each run consisted of one cold and three heated chains. The NJ, ML, and BI analyses were performed with MEGA [63], PhyML [64], and MrBayes [65], respectively.

### Histology and *in situ* hybridization

The hagfish embryos and adult specimens were fixed by immersion in Serra’s fixative, processed for paraffin sectioning by standard methods, and sectioned to 6–10 μm. A single section was place on a glass slide, and the adjacent sections were used for haematoxylin and eosin staining and *in situ* hybridization. The probes were prepared and *in situ* hybridizations were conducted based on previous reports [30,66]. *In situ* hybridization was performed in a Ventana automated machine (Roche Diagnostics, Japan). To detect the signals, a BlueMap NBT/BCIP substrate kit was used, and the samples were counterstained with a nuclear Fast Red equivalent reagent, ISH Red (Roche Diagnostics). We performed several pilot *in situ* hybridization experiments using the olfactory epithelial tissues of the adult hagfish, because the olfactory epithelium is known to express several *Dlx* genes in mammals [67,68] (Additional file 5). The research followed internationally recognized guidelines. We received ethical approval from RIKEN Kobe Institute Safety Center.

## Competing interests

The authors declare that they have no competing interests.

## Authors’ contributions

SF conducted intensive molecular cloning of the *Dlx* genes and *in situ* hybridization. YO and KGO collected the samples and maintained the aquarium tank. KGO and S. Kuraku analysed the molecular sequence data. KGO wrote the first draft of the manuscript. KGO, S. Kuraku, and S. Kuratani contributed to the writing of the final version of the manuscript. All authors discussed the results and commented on the manuscript. All authors read and approved the final manuscript.

## Authors’ information

SF is a technical assistant and YO is a doctoral student in the Laboratory for Evolutionary Morphology at the Center for Developmental Biology (CDB). KGO initiated this study in the same laboratory but now holds an independent position at the Yilan Marine Research Station, ICOB, Academia Sinica in Taiwan. S. Kuraku is the leader of the Genome Resource and Analysis Unit at CDB. S. Kuratani is the director of the Laboratory for Evolutionary Morphology at CDB.

## Supplementary Material

Additional file 1 **Multiple alignment of the conserved regions of the *Dlx *genes. **Predicted amino acid sequences encoded by the *Dlx* genes of *E. burgeri* and various other species. Seven hagfish, six lampreys, 49 gnathostomes, and six tunicate Dlx proteins were aligned by CLUSTALW [62] and visual inspections. Identical sites are boxed. The stop codon site of the hagfish *Dlx* pseudogene is indicated by asterisk and arrowhead. Accession numbers are as follows: *Astyanax mexicanus* Dlx2a [ABG89858.1]; *Astyanax mexicanus* Dlx2b [ABG89859.1]; *Ciona intestinalis* Dlx homologue [NP_001027821, NP_001027672, NP_001027820]; *Danio rerio* Dlx1a, NP_571380.1; *Danio rerio* Dlx2a [CAP19546.1]; *Danio rerio* Dlx2b [NP_571372.1]; *Danio rerio* Dlx3b [NP_571397.2]; *Danio rerio* Dlx4a [NP_571375.1]; *Danio rerio* Dlx5a [AAH83280.1]; *Danio rerio* Dlx6a [NP_571398.1]; *Eptatretus burgeri* Dlx1/4/6A [AB679710]; *Eptatretus burgeri* Dlx1/4/6B [AB679711]; *Eptatretus burgeri* Dlx1/4/6C [AB679712]; *Eptatretus burgeri* Dlx2/3/5A [AB679713]; *Eptatretus burgeri* Dlx2/3/5B [AB679714]; *Eptatretus burgeri* Dlx2/3/5C [AB679715]; *Eptatretus burgeri* Dlx pseudo [AB679716]; *Gallus gallus* Dlx1 [NP_001039307.2]; *Gallus gallus* Dlx3 [NP_990135.1]; *Gallus gallus* Dlx5 [NP_989490.1]; *Gallus gallus* Dlx5 [NP_989490.1]; *Gallus gallus* Dlx6 [NP_001074359.1]; *Homo sapiens* Dlx1 [NP_835221.2]; *Homo sapiens* Dlx2 [NP_004396.1]; *Homo sapiens* Dlx3 [NP_005211.1]; *Homo sapiens* Dlx4 [NP_612138.1]; *Homo sapiens* Dlx5 [NP_005212.1]; *Homo sapiens* Dlx6 [NP_005213.2]; *Lethenteron japonicum* DlxA [AB292628]; *Lethenteron japonicum* DlxB [AB292629]; *Lethenteron japonicum* DlxC [AB292630]; *Lethenteron japonicum* DlxD [AB048759]; *Lethenteron japonicum* DlxE [AB048759]; *Lethenteron japonicum* DlxF [AB292633]; *Monodelphis domestica* Dlx1 [XP_001368011.1]; *Monodelphis domestica* Dlx2 [XP_001368046.1]; *Monodelphis domestica* Dlx3 [XP_001367695.1]; *Monodelphis domestica* Dlx5 [XP_001363081.1]; *Monodelphis domestica* Dlx6 [XP_001363167.1]; *Mus musculus* Dlx1 [NP_034183.1]; *Mus musculus* Dlx2 [NP_034184]; *Mus musculus* Dlx3 [NP_034185.1]; *Mus musculus* Dlx4 [NP_031893.3]; *Mus musculus* Dlx5 [NP_034186.2]; *Mus musculus* Dlx6 [NP_034187]; *Notophthalmus viridescens* Dlx3 [P53770.1]; *Oikopleura dioica* Dlx homologues, [AAW24001, AAW24002, AAV73846]; *Ornithorhynchus anatinus* Dlx2 [XP_001514642.1]; *Oryzias latipes* Dlx2a [NP_001098290.1]; *Petromyzon marinus* DlxA [AAG41495.1]; *Petromyzon marinus* DlxB [AAG41496.1]; *Petromyzon marinus* DlxC [AAG41497.1]; *Petromyzon marinus* DlxD [AAG41498.1]; *Salmo salar* Dlx5a [NP_001134142.1]; *Salmo salar* Dlx5a [NP_001134142.1]; *Synodontis multipunctatus* Dlx2a [ABG89865.1]; *Synodontis multipunctatus* Dlx2b [ABG89866.1]; *Taeniopygia guttata* Dlx1 [XP_002198787.1]; *Taeniopygia guttata* Dlx2 [XP_002196070.1]; *Taeniopygia guttata* Dlx6 [XP_002197360.1]; *Triakis semifaciata* Dlx1 [AAV85983.1]; *Triakis semifaciata* Dlx2 [AAV85984.1]; *Triakis semifaciata* Dlx3 [AAV85985.1]; *Triakis semifaciata* Dlx4 [AAV85986.1]; *Triakis semifaciata* Dlx5 [AAV85987.1]; *Triakis semifaciata* Dlx6 [AAV85988.1]; *Xenopus tropicalis* Dlx1 [NP_001093727.1]; *Xenopus tropicalis* Dlx2 [NP_001008061.1]; *Xenopus tropicalis* Dlx3 [NP_001025566.1]; *Xenopus tropicalis* Dlx5 [NP_001004778.1]; *Xenopus tropicalis* Dlx5 [NP_001004778.1]. Click here for file

Additional file 2 **Unrooted molecular phylogenetic trees including the hagfish *Dlx *genes. **The topologies and branch lengths were calculated with the neighbour-joining method. (A) Phylogenetic tree of *Dlx1/4/6* (based on 155 amino acid sites). (B) Phylogenetic tree of *Dlx2/3/5* (based on 106 amino acid sites). Bootstrap probabilities (NJ and ML) greater than 50 and posterior probabilities (BI) greater than 0.5 are shown at each internal branch supported by all three phylogenetic trees, as the left, middle, and right numbers, respectively. The clades of the cyclostome *Dlx* genes are indicated with bold lines. The hagfish genes are shown in white letters in a black box. Click here for file

Additional file 3 **The expression patterns of the *Dlx *genes in middle- and late-pharyngula embryos of *E. burgeri*. **The sections of the middle-pharyngula embryos (A–H’) and the late-pharyngula embryos (I–X’) were derived from the embryos in Figure 1. Higher magnification of cranial cartilaginous tissues (*trab* and *d.l.b*) and inner ear in the late-pharyngeal embryos are shown in the third (J’–P’) and fifth rows (Q’–X’), respectively. Haematoxylin- and eosin-stained sections (A, I, I’, Q, and Q’) were obtained from the same level as the sections used for *in situ* hybridization with DIG-labelled probes for *EbDlx1/4/6A* (B, J, J’, R, and R’), *EbDlx1/4/6B* (C, K, K’, S, and S’), *EbDlx1/4/6C* (D, L, L’, T, and T’), *EbDlx2/3/5A* (E, M, M’, U, and U’), *EbDlx2/3/5B* (F, N, N’, V, and V’), *EbDlx2/3/5C* (G, O, O’, W, and W’), and *EbDlxΨ* (H, P, P’, X, and X’). The sections for *in situ* hybridization were counterstained with ISH Red. The signals from the probes in the mesenchymal cells (B, C, G, and O) and the cartilaginous primordium (N’) are indicated by arrowheads. Abbreviations: *aud*, auditory capsule; *aud. c*, auditory cartilage; *c. bas*, basal cartilage; *coe*, coelom; *hb*, hindbrain; *d. l. b*, dorsal longitudinal bar; *ex. pq*, extrapalato-quadrate; *nchh*, notochordal hook; *nt*, notochord; *ot*, otic vesicles; *ph*, pharynx; *pp*, pharyngeal pouch; *t. cl*, tendon of the clavatus muscle; *t. vag*, tendon of the vagina of the clavatus; *trab*, trabecular; *v. br. b*, ventral branchial bar; VII, facialis nerve; VIII, saccularis nerve. Scale bars, 100 μm. Click here for file

Additional file 4 List of primer sets. Click here for file

Additional file 5 **Comparison of the expression patterns of the *Dlx *genes in the adult olfactory epithelium of *E. burgeri*. **Haematoxylin- and eosin-stained sections (A) were obtained from the same level as the sections used for *in situ* hybridization with DIG-labelled probes for *EbDlx1/4/6A* (B), *EbDlx1/4/6B* (C), *EbDlx1/4/6C* (D), *EbDlx2/3/5A* (E), *EbDlx2/3/5B* (F), *EbDlx2/3/5C* (G), and *EbDlxΨ* (H). The sections for *in situ* hybridization were counterstained with ISH Red. All the genes were expressed in the olfactory epithelium. *EbDlx1/4/6A*, *EbDlx2/3/5B, and EbDlx2/3/5C* were detected in a few cells across the entire region of the olfactory epithelium. Strongly *EbDlx1/4/6C*-positive cells tended to be located at the levels of the non-basal olfactory epithelium cells. *EbDlx2/3/5A* showed strong expression in the basal cells. Subtle expression of *EbDlx1/4/6B* was detected in the entire region of the olfactory epithelium. *EbDlxΨ* showed a broad expression pattern in the basal cells and olfactory epithelium. The layers in which the probe signals were detected are indicated by brackets on the left side of the panels (B–H). Abbreviations: *bc*, basal cells; *oe*, olfactory epithelium. Scale bar, 100 μm. Click here for file
