# Supplementary figures and images for: Non-parsimonious evolution of hagfish Dlx genes
Source: BMC Evol Biol. 2013 Jan 19;13:15. doi: 10.1186/1471-2148-13-15 (PMC3552724; doi:10.1186/1471-2148-13-15)

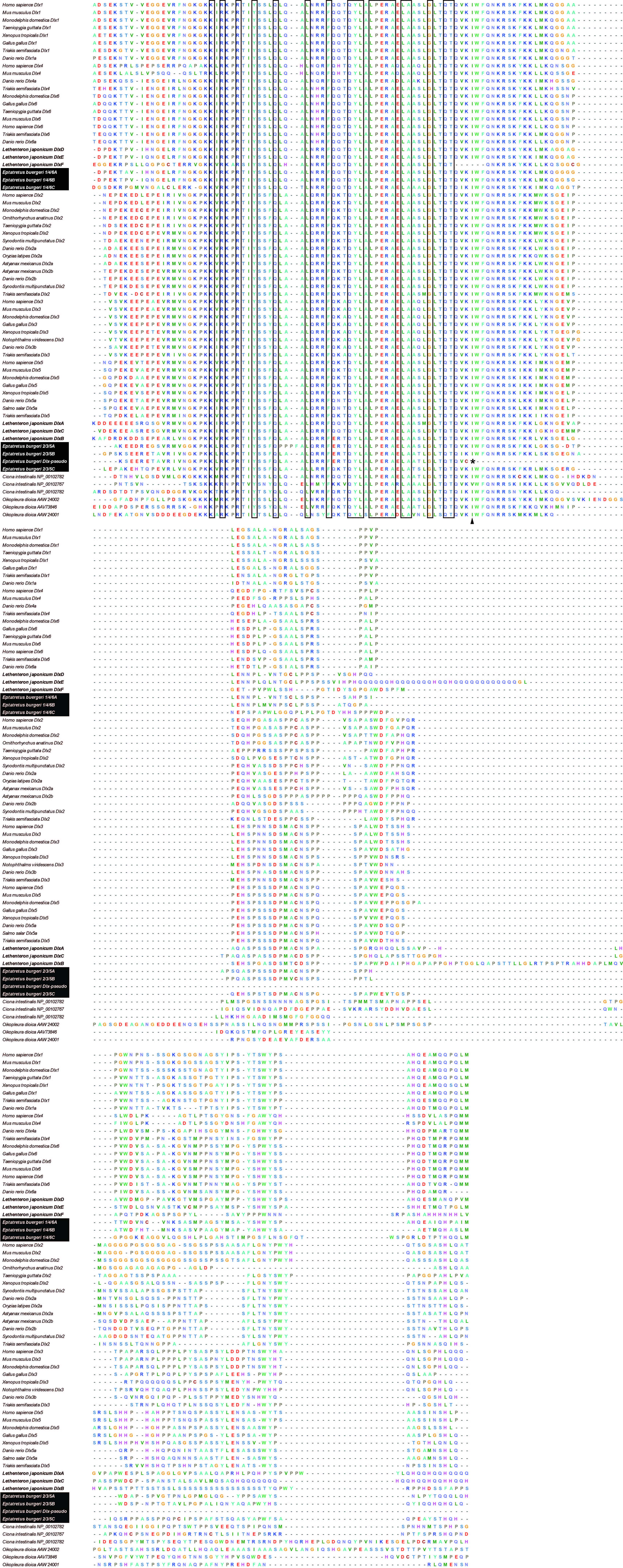

Supplement: Additional file 1 — Multiple alignment of the conserved regions of the Dlx genes. Predicted amino acid sequences encoded by the Dlx genes of E. burgeri and various other species. Seven hagfish, six lampreys, 49 gnathostomes, and six tunicate Dlx proteins were aligned by CLUSTALW [62] and visual inspections. Identical sites are boxed. The stop codon site of the hagfish Dlx pseudogene is indicated by asterisk and arrowhead. Accession numbers are as follows: Astyanax mexicanus Dlx2a [ABG89858.1]; Astyanax mexicanus Dlx2b [ABG89859.1]; Ciona intestinalis Dlx homologue [NP_001027821, NP_001027672, NP_001027820]; Danio rerio Dlx1a, NP_571380.1; Danio rerio Dlx2a [CAP19546.1]; Danio rerio Dlx2b [NP_571372.1]; Danio rerio Dlx3b [NP_571397.2]; Danio rerio Dlx4a [NP_571375.1]; Danio rerio Dlx5a [AAH83280.1]; Danio rerio Dlx6a [NP_571398.1]; Eptatretus burgeri Dlx1/4/6A [AB679710]; Eptatretus burgeri Dlx1/4/6B [AB679711]; Eptatretus burgeri Dlx1/4/6C [AB679712]; Eptatretus burgeri Dlx2/3/5A [AB679713]; Eptatretus burgeri Dlx2/3/5B [AB679714]; Eptatretus burgeri Dlx2/3/5C [AB679715]; Eptatretus burgeri Dlx pseudo [AB679716]; Gallus gallus Dlx1 [NP_001039307.2]; Gallus gallus Dlx3 [NP_990135.1]; Gallus gallus Dlx5 [NP_989490.1]; Gallus gallus Dlx5 [NP_989490.1]; Gallus gallus Dlx6 [NP_001074359.1]; Homo sapiens Dlx1 [NP_835221.2]; Homo sapiens Dlx2 [NP_004396.1]; Homo sapiens Dlx3 [NP_005211.1]; Homo sapiens Dlx4 [NP_612138.1]; Homo sapiens Dlx5 [NP_005212.1]; Homo sapiens Dlx6 [NP_005213.2]; Lethenteron japonicum DlxA [AB292628]; Lethenteron japonicum DlxB [AB292629]; Lethenteron japonicum DlxC [AB292630]; Lethenteron japonicum DlxD [AB048759]; Lethenteron japonicum DlxE [AB048759]; Lethenteron japonicum DlxF [AB292633]; Monodelphis domestica Dlx1 [XP_001368011.1]; Monodelphis domestica Dlx2 [XP_001368046.1]; Monodelphis domestica Dlx3 [XP_001367695.1]; Monodelphis domestica Dlx5 [XP_001363081.1]; Monodelphis domestica Dlx6 [XP_001363167.1]; Mus musculus Dlx1 [NP_034183.1]; Mus musculus Dlx2 [NP_0 [file 1471-2148-13-15-S1.jpeg]

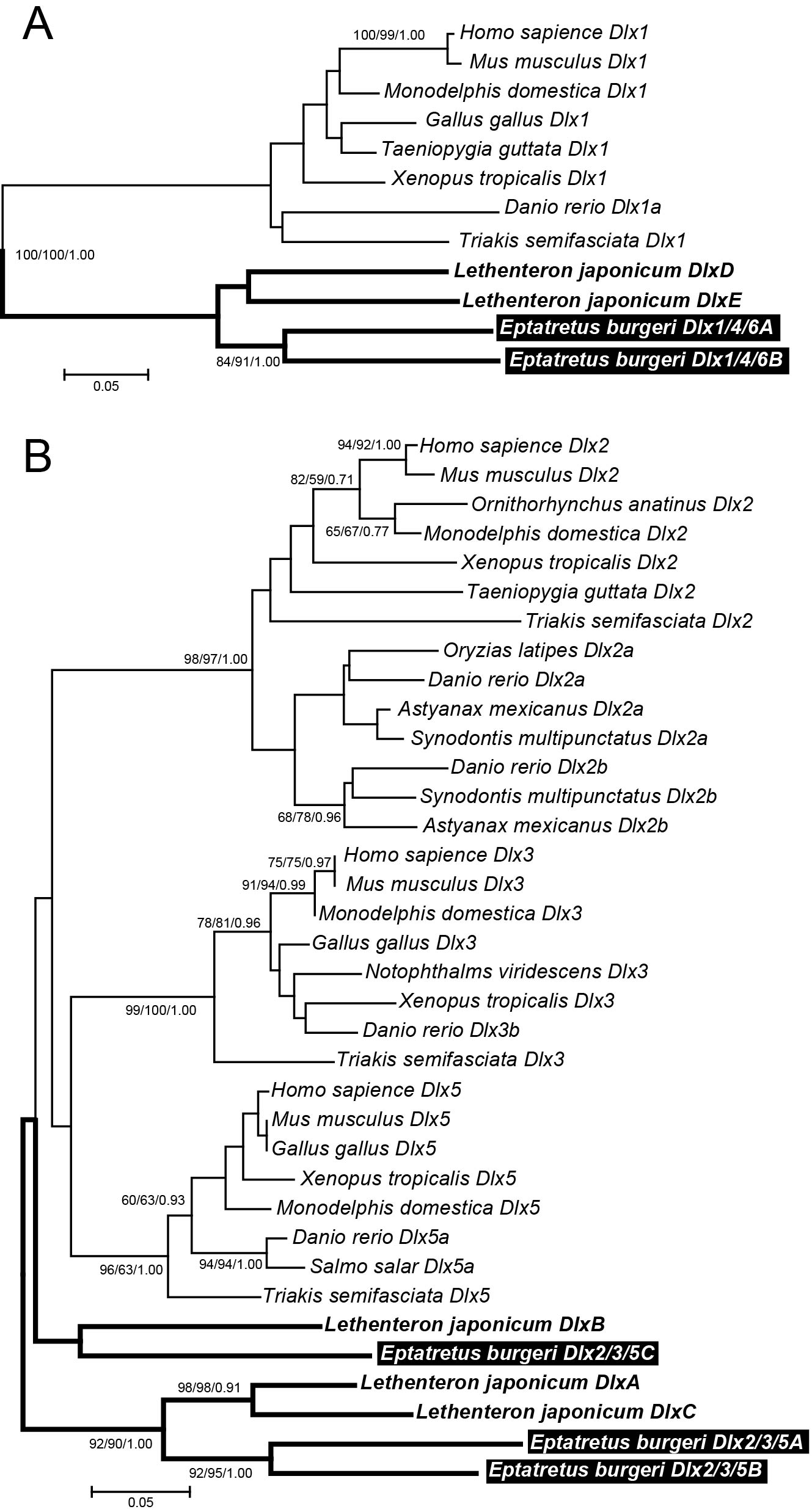

Supplement: Additional file 2 — Unrooted molecular phylogenetic trees including the hagfish Dlx genes. The topologies and branch lengths were calculated with the neighbour-joining method. (A) Phylogenetic tree of Dlx1/4/6 (based on 155 amino acid sites). (B) Phylogenetic tree of Dlx2/3/5 (based on 106 amino acid sites). Bootstrap probabilities (NJ and ML) greater than 50 and posterior probabilities (BI) greater than 0.5 are shown at each internal branch supported by all three phylogenetic trees, as the left, middle, and right numbers, respectively. The clades of the cyclostome Dlx genes are indicated with bold lines. The hagfish genes are shown in white letters in a black box. [file 1471-2148-13-15-S2.jpeg]

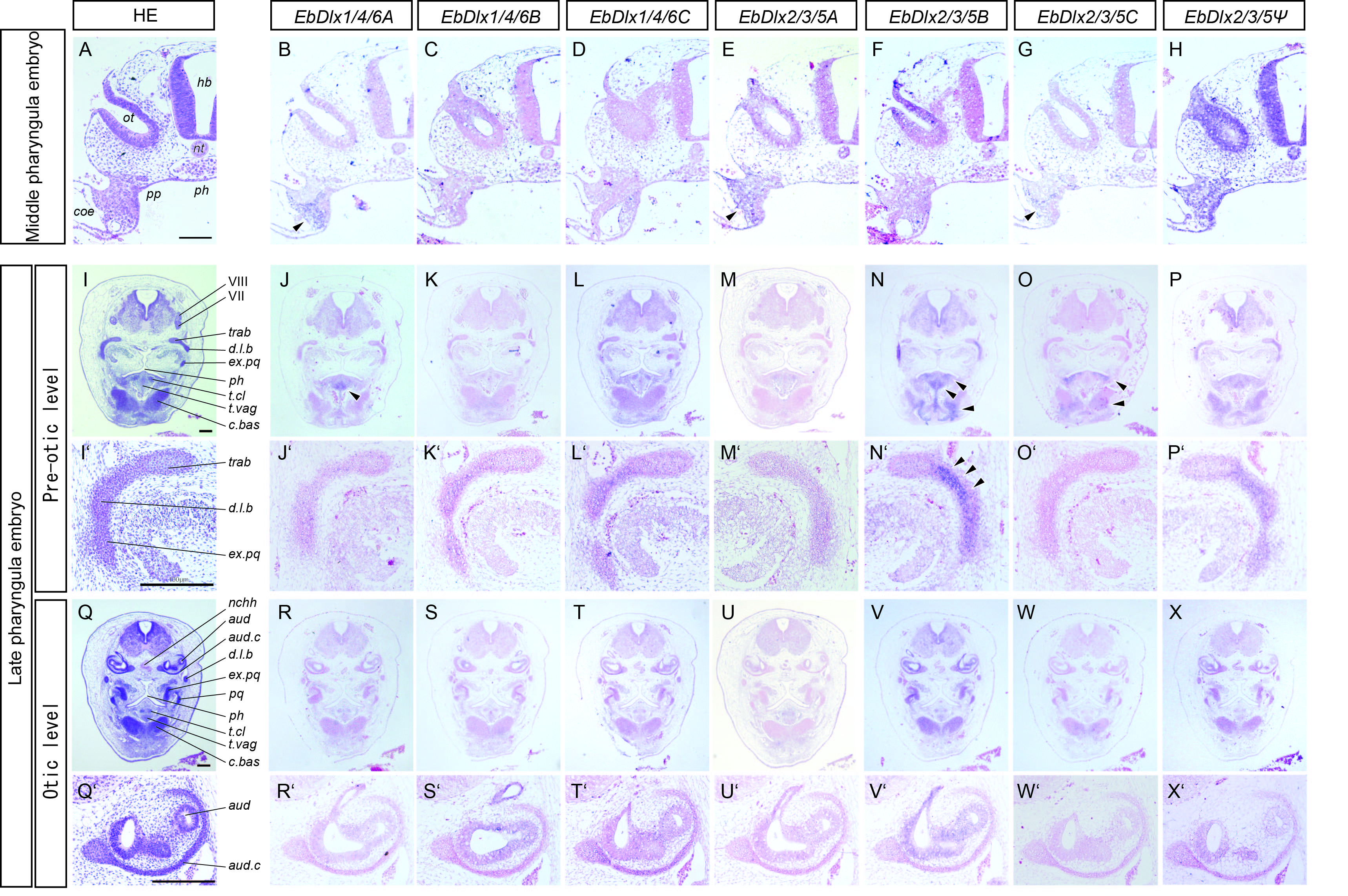

Supplement: Additional file 3 — The expression patterns of the Dlx genes in middle- and late-pharyngula embryos of E. burgeri. The sections of the middle-pharyngula embryos (A–H’) and the late-pharyngula embryos (I–X’) were derived from the embryos in Figure 1. Higher magnification of cranial cartilaginous tissues (trab and d.l.b) and inner ear in the late-pharyngeal embryos are shown in the third (J’–P’) and fifth rows (Q’–X’), respectively. Haematoxylin- and eosin-stained sections (A, I, I’, Q, and Q’) were obtained from the same level as the sections used for in situ hybridization with DIG-labelled probes for EbDlx1/4/6A (B, J, J’, R, and R’), EbDlx1/4/6B (C, K, K’, S, and S’), EbDlx1/4/6C (D, L, L’, T, and T’), EbDlx2/3/5A (E, M, M’, U, and U’), EbDlx2/3/5B (F, N, N’, V, and V’), EbDlx2/3/5C (G, O, O’, W, and W’), and EbDlxΨ (H, P, P’, X, and X’). The sections for in situ hybridization were counterstained with ISH Red. The signals from the probes in the mesenchymal cells (B, C, G, and O) and the cartilaginous primordium (N’) are indicated by arrowheads. Abbreviations: aud, auditory capsule; aud. c, auditory cartilage; c. bas, basal cartilage; coe, coelom; hb, hindbrain; d. l. b, dorsal longitudinal bar; ex. pq, extrapalato-quadrate; nchh, notochordal hook; nt, notochord; ot, otic vesicles; ph, pharynx; pp, pharyngeal pouch; t. cl, tendon of the clavatus muscle; t. vag, tendon of the vagina of the clavatus; trab, trabecular; v. br. b, ventral branchial bar; VII, facialis nerve; VIII, saccularis nerve. Scale bars, 100 μm. [file 1471-2148-13-15-S3.jpeg]

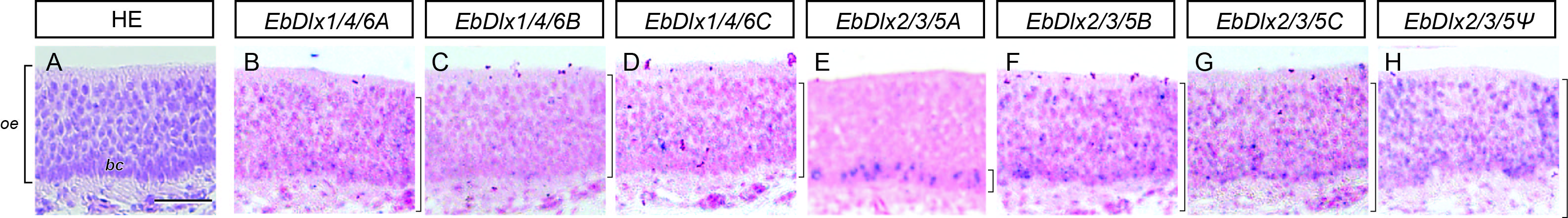

Supplement: Additional file 5 — Comparison of the expression patterns of the Dlx genes in the adult olfactory epithelium of E. burgeri. Haematoxylin- and eosin-stained sections (A) were obtained from the same level as the sections used for in situ hybridization with DIG-labelled probes for EbDlx1/4/6A (B), EbDlx1/4/6B (C), EbDlx1/4/6C (D), EbDlx2/3/5A (E), EbDlx2/3/5B (F), EbDlx2/3/5C (G), and EbDlxΨ (H). The sections for in situ hybridization were counterstained with ISH Red. All the genes were expressed in the olfactory epithelium. EbDlx1/4/6A, EbDlx2/3/5B, and EbDlx2/3/5C were detected in a few cells across the entire region of the olfactory epithelium. Strongly EbDlx1/4/6C-positive cells tended to be located at the levels of the non-basal olfactory epithelium cells. EbDlx2/3/5A showed strong expression in the basal cells. Subtle expression of EbDlx1/4/6B was detected in the entire region of the olfactory epithelium. EbDlxΨ showed a broad expression pattern in the basal cells and olfactory epithelium. The layers in which the probe signals were detected are indicated by brackets on the left side of the panels (B–H). Abbreviations: bc, basal cells; oe, olfactory epithelium. Scale bar, 100 μm. [file 1471-2148-13-15-S5.jpeg]
